# Quantification of PRL/Stat5 signaling with a novel pGL4-CISH reporter

**DOI:** 10.1186/1472-6750-8-11

**Published:** 2008-02-06

**Authors:** Feng Fang, Giovanni Antico, Jiamao Zheng, Charles V Clevenger

**Affiliations:** 1 Department of Pathology, Northwestern University, Chicago, Illinois 60611 USA

## Abstract

**Background:**

Elevations of serum prolactin (PRL) are associated with an increased risk for breast cancer. PRL signaling through its prolactin receptor (PRLr) involves the Jak2/Stat5 pathway. Luciferase-based reporter assays have been widely used to evaluate the activity of this pathway. However, the existing reporters are often not sensitive enough to monitor the effect of PRL in this pathway.

**Results:**

In this study, a new biologically relevant reporter, pGL4-CISH, was generated to study the PRL/Jak2/Stat5 signaling pathway. The sensitivity of pGL4-CISH to detect PRL was superior to that of several other commonly utilized Stat5-responsive reporters. Interestingly, the enhanced function pGL4-CISH was restricted to the estrogen receptor positive (ER+) human breast cancer cell lines T47D and MCF7, but not in the ER-MDA-231, BT-474, or MCF10A cell lines. Overexpression of Stat5 further enhanced the effect of PRL on pGL4-CISH.

**Conclusion:**

These studies demonstrate that pGL4-CISH is a novel and sensitive reporter for assessing the activity of the PRL/Stat5 signaling pathway in the ER+ human breast cancer cells.

## Background

Prolactin (PRL) is a 22 kDa hormone that stimulates the growth and differentiation of mammary epithelium, and initiates and maintains lactation [1-3]. PRL serum levels range from 5–20 ng/ml in blood in non-pregnant humans and up to 200 ng/ml in pregnant females. The lactogenic actions of PRL are mediated by binding to its receptor (PRLr), an event that activates several proximal PRLr signaling cascades including Jak2-Stat5 [4-8]. PRL-induced autophosphorylation of Jak2 results in the activation of this tyrosine kinase, and its subsequent phosphorylation of both the PRLr and the PRLr-associated, latent transcription factor Stat5. Following phosphorylation, Stat5 is released from the PRLr, self-dimerizes and is translocated into the nucleus [6,9-11], where it binds to gene promoter sequences containing Stat5-binding elements, resulting in the induction of gene expression such as Cytokine Inducible SH2-containing protein (CISH or CIS), β-casein, c-Myc, and cyclin D1 [9,12,13].

The Jak2/Stat5 pathway is negatively regulated by a feedback loop through the suppressors of cytokine signaling [13,14], a family of related proteins that includes CISH. The induction of CISH gene expression results in the binding of CISH to the phosphorylated PRLr, which in turn prevents the subsequent binding of Stat5 to PRLr, hence attenuating the PRL/Stat5 signaling pathway [15]. Interestingly, while blocking Stat5 activation CISH does not stop continued Jak2 and MAPK activity. This observation, coupled with the detection of elevated expression of CISH protein in primary human breast cancers, has led to the hypothesis that PRL-induced CISH expression may facilitate the pathogenesis of breast cancer by enhancing cell proliferation triggered by Jak2/MAPK activity at the expense of cell differentiation mediated by Stat5 [16].

Luciferase-reporter assays are widely used to monitor the cellular events related to transduction and gene expression regulated by specific signaling cascades, such as PRL/Jak2/Stat5 pathway. There are several reporter construct used to study Stat5 activity, such as the LHRE-TK-luc [17], cyclin D1Δ-944 [12,18,19], β-casein-344 [20], and β-casein-2300 [21]. However, in breast cancer cells these existing reporter constructs have proven to be relatively insensitive to the effects of PRL, requiring supra-physiologic concentrations of PRL (>200 ng/ml) to detect reporter induction. To generate a more PRL-sensitive reporter construct, our analysis of the literature suggested that the PRL-responsive CISH promoter could be an optimal candidate as it contains four Stat5 binding sites, and is rapidly activated after PRL stimulation [22]. Cloning of the promoter region of the CISH gene into the improved pGL4.10 luciferase reporter construct resulted in a highly sensitive, PRL-responsive reporter that should be of widespread utility in examining PRL/Stat5 pathway in ER+ human breast cancer cells.

## Methods

### Cell lines, reagents and vectors

The breast cancer cell lines T47D, MCF7, BT-474, MDA-231 and the non-tumorigenic epithelial cell line MCF10A from American Type Culture Collection (ATCC) were maintained in Dulbecco's modified Eagle's medium (DMEM) or the similar growth medium supplemented with 10% fetal bovine serum (FBS) and penicillin/streptomycin (Pen/Strep 50 ug/ml) in a humidified atmosphere of 5% CO_2 _at 37°C. Human recombinant prolactin was obtained from Dr. Michael Hodsdon (Yale University) [23]. Transfection reagents Fugene HD (Roche, Indianapolis, IN) for MCF7 cells and Lipofactamine 2000 (Invitrogen, Casbad, CA) for all other cell lines were used for transfection. Vectors Renilla luciferase reporter pGL4.73 (Promega), pGL4.10 (Promega), pGL4-CISH (this study, description below), pEF-PRLr [24], and pEF V5/His A (Invitrogen) were used for transfection.

### Reporter construction

The CISH promoter region (-1034 to +1) was PCR amplified using the primers olg104 5'CCGCCC CAACCTCTATCA-3' and olg110 5'-GGCC**AAGCTT**ACTGAGAGGCAGTGGCG CGGACCGCC-3' (the bold sequence is a HindIII restriction site) using HiFi PCR kit (Invitrogen). For the vector, a promoter-less pGL4 luciferase expression construct (Promega, Madison, WI) was utilized. The EcoRV and HindIII digested pGL4.10 reporter and the HindIII digested PCR product were ligated and transformed into TOP10 *E coli *cells. The construct was confirmed by sequencing using primer olg18 (5'-CCGTCTTCGAGTGGGTAGAAT-3') and RVprimer 3 (Promega). The generated reporter was termed pGL4-CISH. The cyclin D1 promoter region was PCR amplified using the olg88 (5'-ATTG**GGTACC**TAAATCCCGGGGGACCCA CT-3', the bold sequence is a KpnI restriction site) and olg89 (5'-CCGG**AAGCTT**GGAGGCTC CAGGACTTTGCA-3', the bold sequence is a HindIII restriction site). The KpnI and HindIII digested PCR product and pGL4.10 reporter backbone were ligated and the construct was named as pGL4-CCND300.

### Transfection and dual luciferase assay

T47D cells were plated in a 24-well plate and grown at 60% confluency for transfection. For luciferase assays, 50 ng of a given reporter construct, 0.5 ng of renilla (pGL4.73 from Promega) and/or 250 ng of pCMV-wtStat5a per well were used for transfection. After transfection, cells were maintained in 500 ul of growth media overnight, and then arrested in 200 ul of serum-free, phenol red-free DMEM for 24 hours followed by PRL treatment for 24 hours. After treatment, cells were lysed in 120 ul of 1 × passive lysis buffer (PLB buffer from Promega) at room temperature for 15 minutes. For dual luciferase assay, 25 ul of lysate was aliquoted into a 96-well plate and subjected to analysis of firefly luciferase (50 ul of LAR II) and renilla (50 ul of stop and glow buffer). Luminescence was read on Victor3 1420 Multilabel Counter (Perkin Elmer, Waltham, MA). All analyses were performed in duplicate, with each experiments performed at least twice.

### Western blot

Forty microliters of cell lysates from luciferase assay were loaded onto 10% SDS-PAGE gel. Proteins were transferred onto PVDF membrane and western blots were performed using 50% skim milk (1:1 ratio of skim milk and 1 × TBS). Antibodies anti-Stat5a (Zymed, 71–2400, 1:2000 dilution), anti-PRLr (Zymed, 35–9200, 1:2000 dilution) and anti-GAPDH (Zymed, 39–8600, 1:2000 dilution) were used for Western blot analysis. Images were obtained using a Fujifilm LAS-3000 image analyzer.

## Results

**Figure 1:**
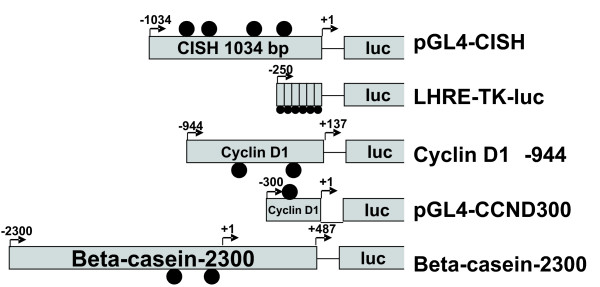
Promoter regions from commonly used reporters to evaluate the PRL/Jak2/Stat5 pathway. Stat5 binding sites are indicated by filled circles.

At present, there are several reporters available for the study of the Stat5 signaling pathway, that includes a synthetic reporter with six-tandem Stat5 responsive elements (LHRE-TK-luc), as well as reporter constructs containing the promoter regions from PRL-responsive genes, namely two cyclin D1 promoter-derived sequences (pGL4-CCND300 and the cyclin D1Δ-944 reporters containing 300 bp and 944 bp of the cyclin D1 promoter), and the rat β-casein promoter sequences (β-casein-344 and β-casein 2300) (Fig 1A and Table 1). All of these reporters commonly carry one or more Stat5 responsive elements and have been widely used to study the PRL-induced activation of Stat5 pathway (Fig. 1A and Table 1). However as noted above, they are relatively insensitive to the effect of PRL.

**Table 1:** Comparison of Reporter Responses to PRL in T47D cells

Reporters	DNA source	Stat5 binding sites	Sensitivity to PRL	References
pGL4-CISH	human	4	+ + +	this work
LHRE-TK-luc	synthetic tandems	6	+ +	[17]
pGL4-CCND300	human	1	+	this work
Cyclin D1Δ-944	human	2	+	[18]
Beta-casein-2300	rat	2	+ +	[21]

(+ + +, most sensitive; + +, sensitive; +, less sensitive in terms of reporter readout)

CISH is a repressor through a negative feedback mechanism in the Jak2/Stat5 pathway [15]. A web-based software (TFsearch) was used to search for Stat5 binding sites in the CISH promoter region, and found four Stat5 binding sites in the proximal region (Fig. 1A). In order to evaluate the response of the human CISH promoter in the context of the Stat5/Jak2 signaling pathway, a 1034 bp fragment (-1034 to +1) was cloned into upstream of a luciferase reporter gene in the promoterless pGL4.10 reporter, resulting in a novel reporter construct termed as pGL4-CISH. Initial use of this reporter in T47D cells revealed that the construct was highly responsive to PRL.

**Figure 2:**
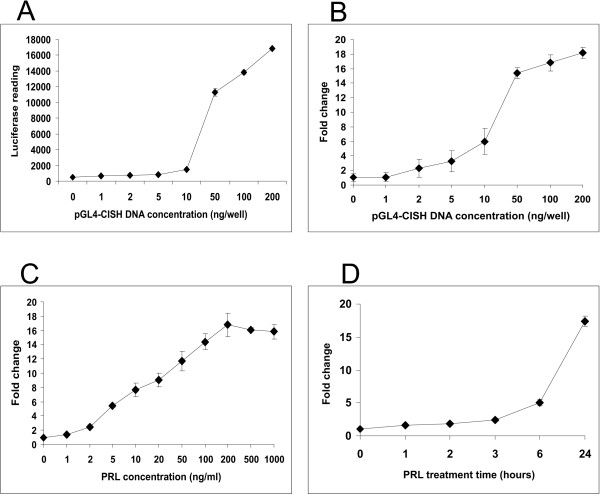
**The dose response and time course of pGL4-CISH to PRL in T47D breast cancer cells.** Following transfection of 1 × 10^5 ^T47D cells with the pGL4-CISH and renilla control reporters, transfectants were cultured in minimal defined medium without FBS for 24 hours followed by 24 hours of PRL stimulation prior to luminescence assay. The pGL4.10 reporter backbone was used to compensate DNA amount used for transfection. A. Luciferase luminescence in PRL-stimulated T47D cells transfected with varying concentrations of pGL4-CISH reporter DNA (PRL concentration, 100 ng/ml). B. Fold changes of normalized PRL-stimulated pGL4-CISH expression versus non-PRL-stimulated T47D transfectants as a function of transfected pGL4-CISH concentration (PRL concentration, 100 ng/ml). C. PRL dose response of expression of pGL4-CISH in T47D transfectants. T47D cells were transfected with 50ng pGL4-CISH/1 × 10^5 ^cells, arrested and treated with PRL (100 ng/ml) for 24 hours. D. Time course of pGL4-CISH expression in the presence of PRL (100 ng/ml).

To evaluate the luciferase signal strength as a function of the quantity of transfected pGL4-CISH reporter, varying concentrations of the pGL4-CISH reporter DNA (0–200 ng/1 × 10^5 ^cells per well in a 24-well plate) were transfected into T47D cells. These studies revealed that 50 ng of pGL4-CISH DNA resulted in a ~50% maximal luminescence signal intensity in the presence of PRL (Fig 2A and 2B). To further optimize the pGL4-CISH reporter assay, the response of this construct to different concentrations of PRL was tested. Results indicated that pGL4-CISH was induced 2-fold with 1 ng/ml of PRL, 8-fold with 10 ng/ml of PRL, saturating at 200 ng/ml of PRL (Fig 2C). Additional time course analysis revealed a relatively rapid 4-fold induction of the pGL4-CISH reporter after as short as 6 hours of PRL treatment (Fig 2D).

**Figure 3:**
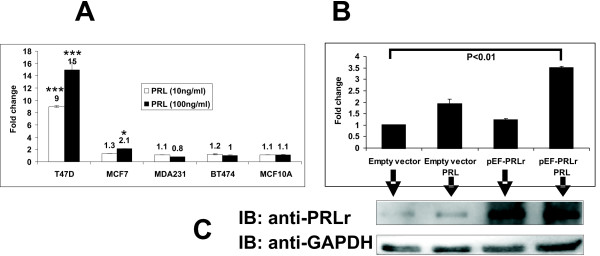
**Expression of pGL4-CISH to PRL and PRLr in different human breast cancer and epithelial cell lines.** Cells were transfected with pGL4-CISH. After transfection, cells were arrested in minimal defined medium without FBS, the transfectants were stimulated with PRL for 24 hours prior to analysis of luminescence. A. Cells lines were transfected with 50 ng pGL4-CISH/1 × 10^5 ^cells. B. MCF7 cells were overexpressed with pEF-PRLr and treated with PRL (200 ng/ml). *, P < 0.05. ***, P < 0.001. C. The low inset is Western blot demonstrating PRLr overexpression level obtained with the various transfectants with the arrows indicating each lane of treatments. Error bars represent SEM; * denotes *P *< 0.05, and *** denotes *P *< 0.001 as compared with no PRL stimulation alone.

The response of the pGL4-CISH in human breast epithelial lines was assessed by its transection into a panel of ER+ and ER- cell lines. The ER+ breast cancer cell lines T47D, MCF7, the ER- MDA-231, BT-474 and the non-tumorigenic epithelial cell line MCF10A were chosen for varying levels of PRLr expression (relative PRLr expression: T47D>MCF7>BT-474≈ MDA-231≈ MCF10A) [25,26]. Results showed that robust expression of pGL4-CISH was obtained in T47D transfectants (Fig 3A). In MCF7 transfectants, pGL4-CISH was increased 2.1 fold following PRL stimulation. No significant increase in expression from the pGL4-CISH reporter was noted following PRL stimulation in the MCF10A, MDA231 or BT474 cell lines (Fig 3A). As the responsiveness of the pGL4-CISH reporter may be related to PRLr expression, the response of this reporter in MCF7 transfectants overexpressing the human PRLr was tested. Overexpression of PRLr enhanced the PRL-induced response of pGL4-CISH in MCF7 cells (Fig 3B and 3C), suggesting that PRLr levels contributed to the responsiveness of the pGL4-CISH expression in MCF7 cells.

**Figure 4:**
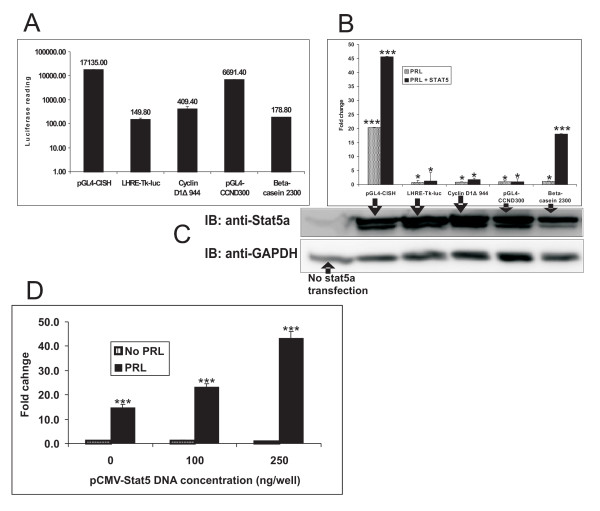
**Comparison of PRL-induced expression obtained from the pGL4-CISH versus other PRL-responsive reporters in T47D transfectants in the presence and absence of overexpressed Stat5.** T47D cells were transfected with vectors and PRL-stimulated as described above. A. The basal luciferase expression of the various PRL-responsive reporters in T47D transfectants in the absence of PRL. B. The response of reporters to PRL in the presence or absence of overexpressed Stat5a in T47D transfectants. C. The low inset is Western blot demonstrating Stat5a overexpression level obtained with the various transfectants with the arrows indicating each lane of treatments. D. Level of transfected Stat5a influences pGL4-CISH expression. Varying concentrations as indicated of Stat5a expression construct were simultaneously transfected with the pGL4-CISH reporter. Error bars represent SEM; * denotes *P *< 0.05, and *** denotes *P *< 0.001 as compared no PRL stimulation alone.

To assess the sensitivity of pGL4-CISH reporter in T47D cells, in contrast to other commonly utilized PRL-responsive expression constructs such as LHRE-TK-luc, Cyclin D1Δ-944, pGL4-CCND300 and β-casein-2300 reporters, direct comparisons of PRL-induced luminescence of T47D transfectants were performed. As shown in Fig 4A, pGL4-CISH had the highest basal luciferase activity (17000 units) compared to LHRE-TK-luc (150 units), beta-casein-2300 (180 units), pGL4-CCND300 (6700 units) and Cyclin D1Δ-944 (410 units) (Fig 4A). Despite this higher level of basal luciferase activity seen with pGL4-CISH, the overall PRL-induced expression from the pGL4-CISH reporter was markedly increased over the other reporter constructs evaluated. The pGL4-CISH luciferase activity was robustly induced by 19-fold following PRL stimulation, while all of the other reporter only constructs showed a 2–3 fold increase in luciferase expression at high physiologic concentration of PRL (100 ng/ml). The effect of increased Stat5a levels on reporter responsiveness was also tested. Results showed that the overexpression of Stat5a also enhanced PRL induction of luciferase activity in all the tested reporters, with pGL4-CISH showing the highest responsiveness (Fig 4B + C). The dose responsiveness of Stat5a on pGL4-CISH was also tested. As shown in Figure 4D, increasing expression of Stat5a did not alter the reporter basal activity in the absence of PRL. However, in the presence of PRL, pGL4-CISH luciferase activity increased as a function of quantity of transfected Stat5a (Fig 4D). Taken together, our data indicate that the responsiveness of the pGL4-CISH reporter is sensitive to both PRLr and Stat5a expression levels.

## Discussion

The versatility and simplicity of the reporter assay is a powerful tool in the fields of biological and pharmaceutical research [27], and there is an ongoing need for highly responsive reporters [28]. Until recently, older reporter vector backbones had spurious transcription factor binding sites resulting in both inaccurate and false-positive results. For the studies presented here, a new pGL4 reporter with decreased cryptic DNA regulatory elements and transcription factor binding sites was used as a backbone for reporter. We constructed a novel pGL4-CISH reporter based on the pGL4 backbone and the Stat5-response-rich-element CISH promoter. The pGL4-CISH DNA dose curve, PRL dose curve and time course parameters were optimized for this reporter. Our results indicated that this reporter had a high sensitivity to PRL in T47D, less so in MCF7, and little to none in ER- cell lines evaluated. As subsequently shown with MCF7 cells, our data would indicate that both PRLr and Stat5a levels contribute to the responsiveness of the pGL4-CISH reporter.

Cell proliferation, as measured by ^3^H-thymidine incorporation, is a sensitive and simple way to test PRL action [29]. In the T47D proliferation assay, maximal stimulation (2.2-fold over basal levels, reaching a plateau) was achieved with 100 ng/ml of PRL treatment [29]. In our study, pGL4-CISH reporter demonstrated a 2-fold induction upon 1 ng/ml of PRL treatment, suggesting this reporter was significantly more sensitive than the thymidine-incorporated proliferation assay to detect breast cancer cell responsiveness to PRL stimulation.

The luciferase induction of pGL4-CISH, LHRE-TK-luc, Cyclin D1Δ-944, pGL4-CCND300 and Beta-casein2300 reporters was also compared in T47D cells. Results indicated that pGL4-CISH reporter was the most responsive to PRL treatment in T47D cells. A reporter termed -404CIS-LUC containing about 500 bp of human CISH promoter has also been reported [30]. However, this reporter demonstrated poor PRL-induced expression in COS7 cells [30]. We reason the different response to PRL of reporters is due to the limited proximal promoter regions utilized, the different backbones used for reporter construct, and cells lines used for reporter assay.

PRL activates the latent cytoplasmic Stat5 by tyrosine phosphorylation and dimerization in PRL/Jak2/Stat5 pathway [31]. The intranuclear phospho-Stat5 then activates the Stat5-responsive-gene expression. We observed that in the absence of PRL, overexpression of Stat5a does not stimulate pGL4-CISH luciferase. In the presence of PRL, overexpression of Stat5 greatly enhanced the effect of PRL on pGL4-CISH reporter (Fig 4D). These data indicate that the activation of pGL4-CISH is influenced by the level of available Stat5a following PRL treatment.

The pGL4-CISH reporter has potential applications in the analysis of the PRL/Stat5 pathway, *CISH *promoter analysis, and biological screening for drug discovery. Indeed, as a novel bioassay for PRL, the pGL4-CISH reporter has a sensitivity approaching that of the venerable rat Nb2 lymphoma cell bioassay for PRL. As a distinct advantage, the PGL4-CISH system now provides a sensitive, species homologous assay for the action of human PRL in human cell lines expressing sufficient levels of PRLr and Stat5. The CISH promoter has four Stat5 binding sites but also include other potential transcriptional binding sites such as Sp1, HSF, c-Myb, and GATA-1. Detailed functional mapping of this promoter to characterize the relative contributions of *cis*-acting elements will improve our understanding of how PRL signaling triggers the response of the *CISH *at the transcriptional level.

## Conclusion

Given its sensitivity to PRL, the pGL4-CISH reporter should be a useful tool in the screening of small compound libraries in the identification of novel PRL/Stat5 inhibitors.

## Authors' contributions

FF carried out pGL4-CISH cloning, Fig 1, Fig 2A, 2B, 2D, 3B, 3C and paper writing. GA carried out Fig 4A, 4B, 4C and 4D. JZ carried out Fig 2C and Fig 3A. CC carried out experimental design, planning and paper correcting. All authors read and approved the final manuscript.
